# Expression profiles and functional prediction of histone acetyltransferases of the MYST family in kidney renal clear cell carcinoma

**DOI:** 10.1186/s12885-023-11076-x

**Published:** 2023-06-26

**Authors:** Fan Liang, Xiangke Li, Xiaoman Shen, Runlei Yang, Chuan Chen

**Affiliations:** 1 School of Basic Medicine, Weifang Medical University, Weifang, 261000 Shandong P.R. China; 2 Institute of Life Science and Green Development, Hebei University, Baoding, 071002 Hebei P.R. China; 3 Key Laboratory of Microbial Diversity Research and Application of Hebei Province, Hebei University, Baoding, 071002 Hebei P.R. China; 4 Engineering Laboratory of Microbial Breeding and Preservation of Hebei Province, Hebei University, Baoding, 071002 Hebei P.R. China

**Keywords:** MYST HATs, Kidney clear cell carcinoma, Prognostic significance, GSEA, Immune infiltrates

## Abstract

**Background:**

Histone acetyltransferases (HATs) of the MYST family are associated with a variety of human cancers. However, the relationship between MYST HATs and their clinical significance in kidney renal clear cell carcinoma (KIRC) has not yet been evaluated.

**Methods:**

The bioinformatics method was used to investigate the expression patterns and prognostic value of MYST HATs. Western blot was used to detect the expression of MYST HATs in KIRC.

**Results:**

The expression levels of MYST HATs except *KAT8* (*KAT5*, *KAT6A*, *KAT6B*, and *KAT7*) were significantly reduced in KIRC tissues compared to normal renal tissues, and the western blot results of the KIRC samples also confirmed the result. Reduced expression levels of MYST HATs except *KAT8* were significantly associated with high tumor grade and advanced TNM stage in KIRC, and showed a significant association with an unfavorable prognosis in patients with KIRC. We also found that the expression levels of MYST HATs were closely related to each other. Subsequently, gene set enrichment analysis showed that the function of *KAT5* was different from that of *KAT6A*, *KAT6B* and *KAT7*. The expression levels of *KAT6A*, *KAT6B* and *KAT7* had significant positive correlations with cancer immune infiltrates such as B cells, CD4^+^ T cells and CD8^+^ T cells.

**Conclusions:**

Our results indicated that MYST HATs, except KAT8, play a beneficial role in KIRC.

**Supplementary Information:**

The online version contains supplementary material available at 10.1186/s12885-023-11076-x.

## Background

Renal cell carcinoma (RCC) has been ranked as the seventh leading cancer type in the developed world [1]. RCC is classified into several histological subtypes such as clear cell renal cell carcinomas, papillary renal cell carcinomas and chromophobe renal cell carcinomas. Among them, KIRC is the most common and lethal subtype of RCC [2]. Surgical and targeted immunotherapy therapies are indispensable treatment options for RCC [3–5]. However, tumor recurrence and drug resistance are the main problems in the treatment of RCC. Exploring predictive biomarkers is very important for the early diagnosis and prognosis of RCC.

The Histone acetyltransferases (HATs) of the MYST family are highly conserved from yeast to humans and function exclusively in multisubunit protein complexes [6]. Their main feature is the existence of a highly conserved MYST domain, which is composed of an acetyl CoA binding motif and a zinc ring. The HATs of the MYST family comprise five members: KAT5 (Tip60), KAT6A (MOZ/MYST3), KAT6B (MORF/MYST4), KAT7 (HBO1/MYST2) and KAT8 (MOF/MYST1) [7]. MYST family HATs can catalyze protein acetylation in both the nucleus and cytoplasm. MYST HATs are involved in various biological functions, including nuclear processes, transcription, DNA damage response, DNA repair, DNA replication, stress response and metabolism [8]. Abnormally expressed MYST HATs are closely associated with some human cancers and neurodegenerative diseases [9, 10].

To date, the significance of the expression of MYST HATs in relation to the clinicopathological and prognostic variables of patients with KIRC has not yet been fully revealed. In this study, we performed an integrative analysis of MYST HATs in KIRC based on the public database.

## Methods

### Gene correlation and prognostic significance analysis in GEPIA

The online database Gene Expression Profiling Interactive Analysis (GEPIA) (http://gepia.cancer-pku.cn/index.html) [11] was used to obtain information on differential expression analysis, gene profiling, correlation analysis, and patient survival analysis of MYST HATs based on TCGA and GTEx data (*P < 0.05, FDR adjustment).

### Gene expression analysis

The correlations of MYST HAT expression with the clinicopathological characteristics of patients with KIRC were determined using the UALCAN database [12] (http://ualcan.path.uab.edu/index.html). Pancancer analysis was performed using the TIMER web server (http://timer.comp-genomics.org/). The statistical significance computed by the Wilcoxon test is annotated by the number of stars (*: *P* value < 0.05; **: *P* value < 0.01; ***: *P* value < 0.001). Immunohistochemical images of cancer and normal tissue were downloaded from Human Protein Atlas (http://www.proteinatlas.org/).

### GSEA

Gene set enrichment analysis (GSEA) is a method for analyzing microarray data of whole genome expression profiles, which compare genes with predefined gene sets. The LinkedOmics online database was used to perform a GSEA analysis [13], which explored the correlation between the potential functions of MYST HAT expression and the pathogenesis of KIRC based on the TCGA and GTEx data (FDR < 0.05) (http://linkedomics.org/login.php).

### Immune infiltrate analysis

The TIMER web server is a comprehensive resource for systematic analysis of immune infiltrates in various types of cancer [14]. The Gene module on the TIMER web was used to explore the correlation between gene expression and abundance of immune infiltrates (https://cistrome.shinyapps.io/timer/).

### Western blot analysis of KIRC tissue samples

KIRC tissue samples and adjacent paired non-cancerous tissues were obtained from 20 KIRC patients. All samples were stored at the Affiliated Hospital of Hebei University. The number of subjects, the age range, the sex ratio, the criteria used for the diagnosis, and the description of any control subjects were shown in Additional File 1. All procedures follow the ethical guidelines for the storage and use of human biological samples. The tissue samples were rapidly frozen in liquid nitrogen and stored at -80 ° C. The Ethics Committee of the Affiliated Hospital of Hebei University approved the present investigation (NO.2020-KY-021). Tissue samples were collected and dissolved in RIPA lysis buffer. After centrifugation, the supernatant was quantified by the Bradford method. 20 µg of the supernatant was subjected to SDS-PAGE and transferred to a PVDF membrane, then incubated with antibodies, KAT5 (ab137518), KAT6B (ab246879) KAT7 (ab190908) (ABCAM, MA, USA), and KAT6A (sc-293,283) (Santa Cruz Biotechnology, MA, USA) (dilution 1:500). Anti-actin antibody (sc-8432) (dilution 1:1000) was used to ensure equal protein loading (Santa Cruz Biotechnology, MA, USA). Anti-rabbit IgG, HRP-linked antibody (sc-2357) and anti-goat IgG, HRP-linked antibody (sc-2354) were used as secondary antibody (dilution 1:1000) (Santa Cruz Biotechnology, MA, USA). Densitometry intensity was calculated using image J software.

### Statistical analysis

The expression results were displayed in the fold change with the P values. Survival curves were made using the Kaplan-Meier method. Spearman rank correlation was performed to investigate the correlation of gene expression. *P* < 0.05 was considered statistically significant.

## Results

### Gene expression profiling and prognostic value in pancancer analysis

**Fig. 1:**
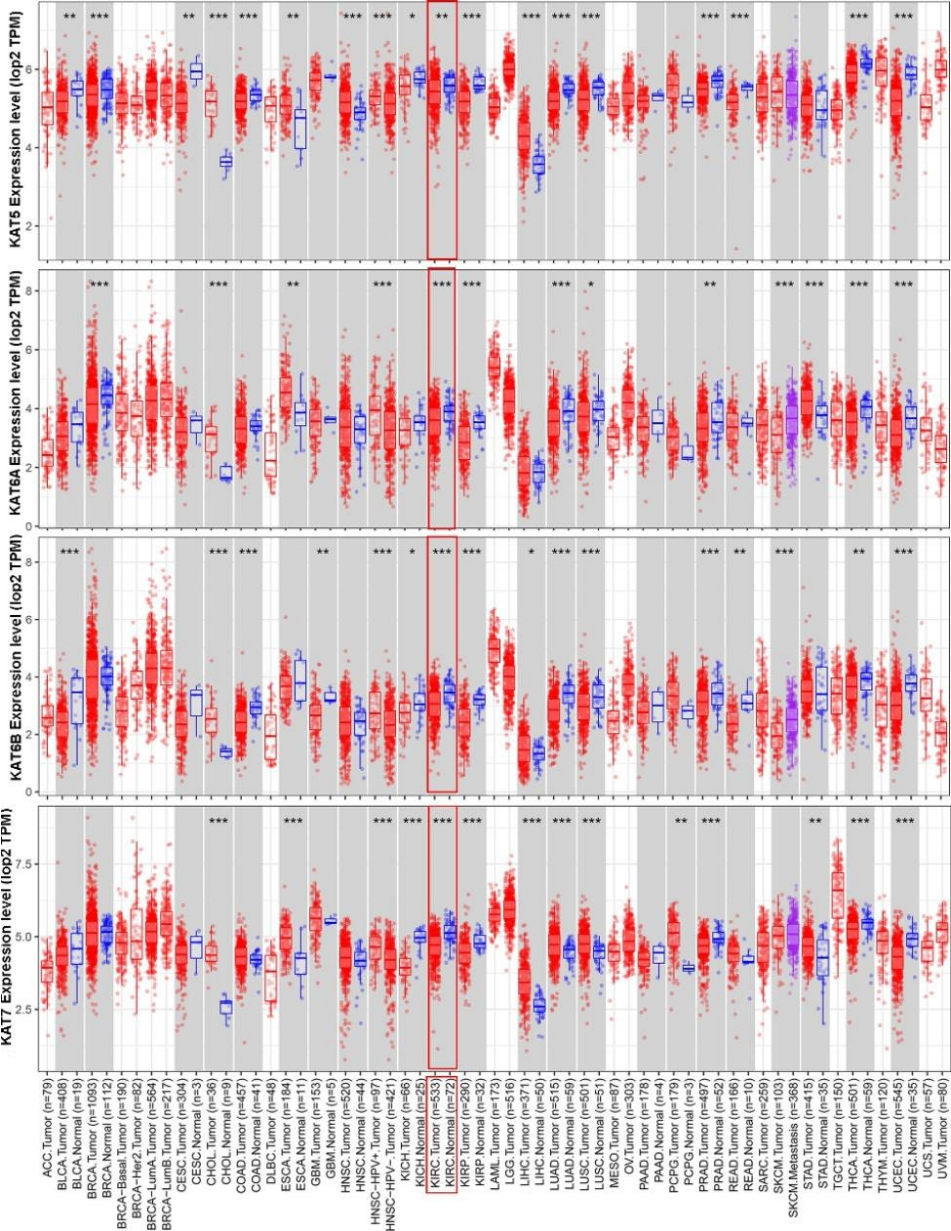
**Expression of MYST HATs in pancancer analysis.** The mRNA levels of MYST HAT in KIRC tumors and adjacent normal tissues were determined using the TIMER database. *, *P* < 0.05; **, *P* < 0.01; ***, *P* < 0.001. Compared to normal tissues

**Fig. 2:**
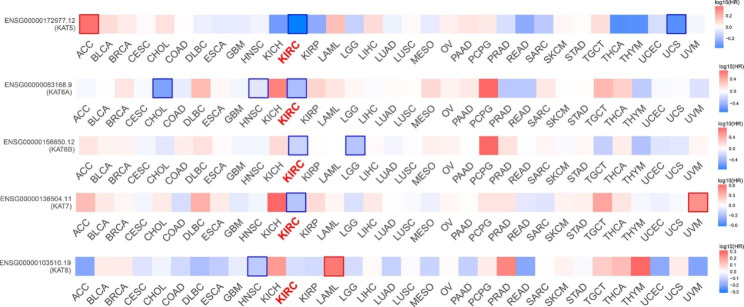
**Summary of the hazard ratios (HR) of MYST HAT expression with overall survival.** The analysis was performed using the TCGA database. Log rank (mantel – Cox) test, *P* < 0.05, FDR adjustment

To determine the expression profile and clinical significance of MYST HATs in different types of cancer, we analyzed the expression of MYST HATs in the TCGA database based on 19 types of cancer. MYST HATs were highly expressed in some cancers but low in others. There was obvious heterogeneity among different cancer types. *KAT5* exhibited significant down-regulation in 10 of the 13 significantly changed cancer types compared to normal tissues, *KAT6A* significantly decreased in 8 of the 11 significantly changed cancer types, *KAT6B* and *KAT7* were significantly down-regulated in 10 cancer types and 6 cancer types, respectively (Fig. 1). The relationship between these genes and survival rate was then analyzed using a log rank test. We found that high expression of MYST HATs except *KAT8* was closely related to an increase in overall survival in patients with KIRC (Fig. 2). In the following study, we will focus on the clinical significance of MYST HATs except KAT8.

### The expression level of MYST HATs in KIRC

**Fig. 3:**
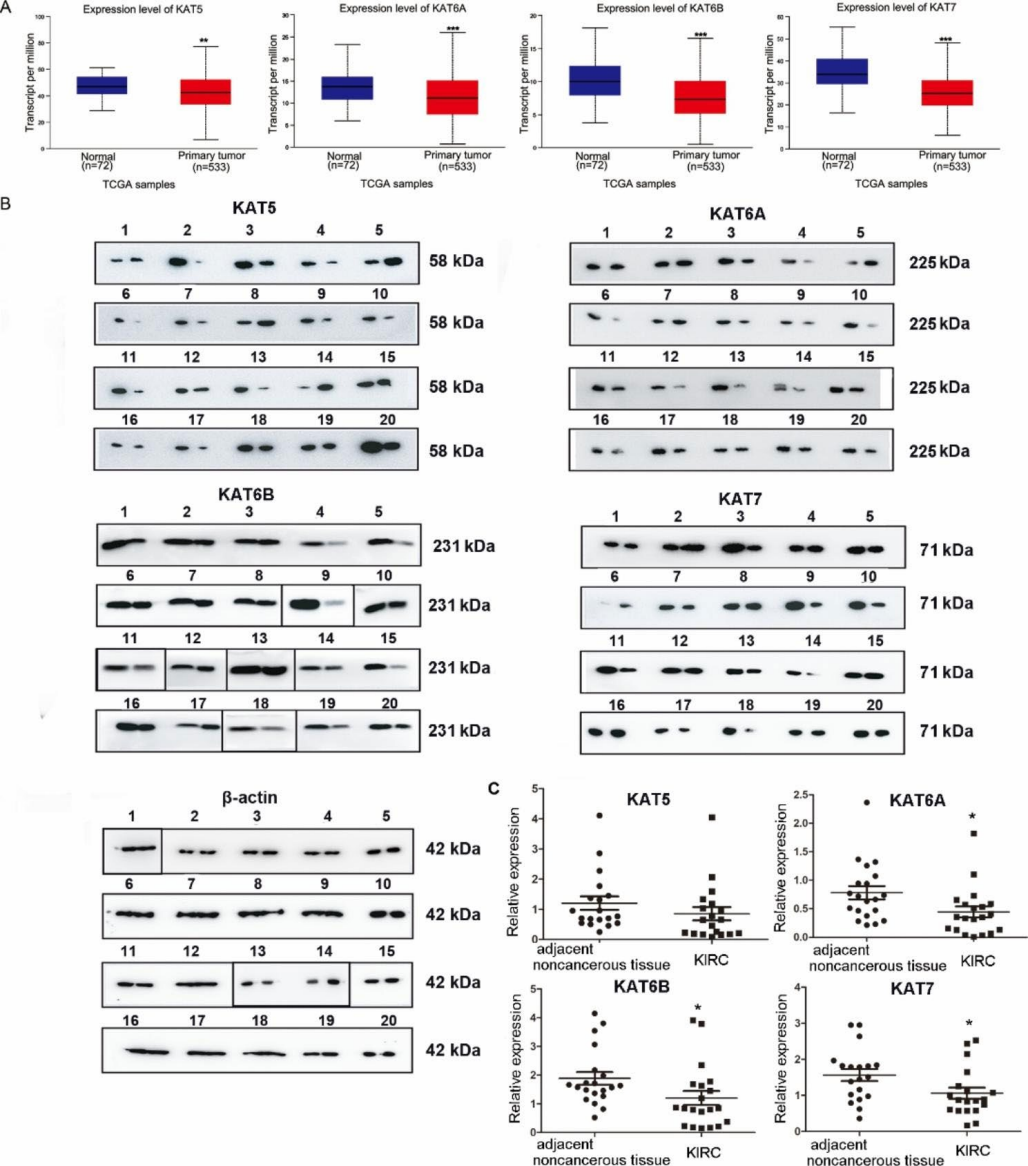
**Expression level of MYST HATs in KIRC.** (**A)** MYST HAT mRNA levels in KIRC patient, **, *P* < 0.01; ***, *P* < 0.001. compared to normal tissues (UALCAN database) (**B)** Western blot analysis of MYST HATs in KIRC. KIRC tissue samples and adjacent non-cancerous tissue samples were obtained from 20 KIRC patients. The left band of each sample was adjacent non-cancerous tissue, and the right band of each sample was KIRC tissue. Actin was used as an internal control. The boxes marked in the figure have been cropped. Original blots were cut prior to hybridization with antibodies (see a detailed explanation in Additional File 2) (**C)** Relative expression analyses using densitometry intensity ratio of western blot bands. Student’s *t*-test, two paired, compared to adjacent non-cancerous tissue *, *P* < 0.05

We analyzed the expression of MYST HATs in KIRC tissues based on TCGA data, and found that MYST HATs significantly decreased in KIRC tissues compared to normal tissues (Fig. 3A). We also investigated the protein expression of 20 clinical samples from KIRC patients using western blot (Fig. 3B). The expression levels of KAT5, KAT6A, KAT6B and KAT7 were compared in paired KIRC tissue and adjacent non-cancerous tissues. We found that MYST HATs decreased in most KIRC tumor tissues (Fig. 3B). Densitometry analysis showed that KAT6A, KAT6B and KAT7 significantly decreased in KIRC tumor tissues (Fig. 3C), but KAT5 in KIRC tumor tissues decreased to some extent, but the change was not significant.

**Fig. 4:**
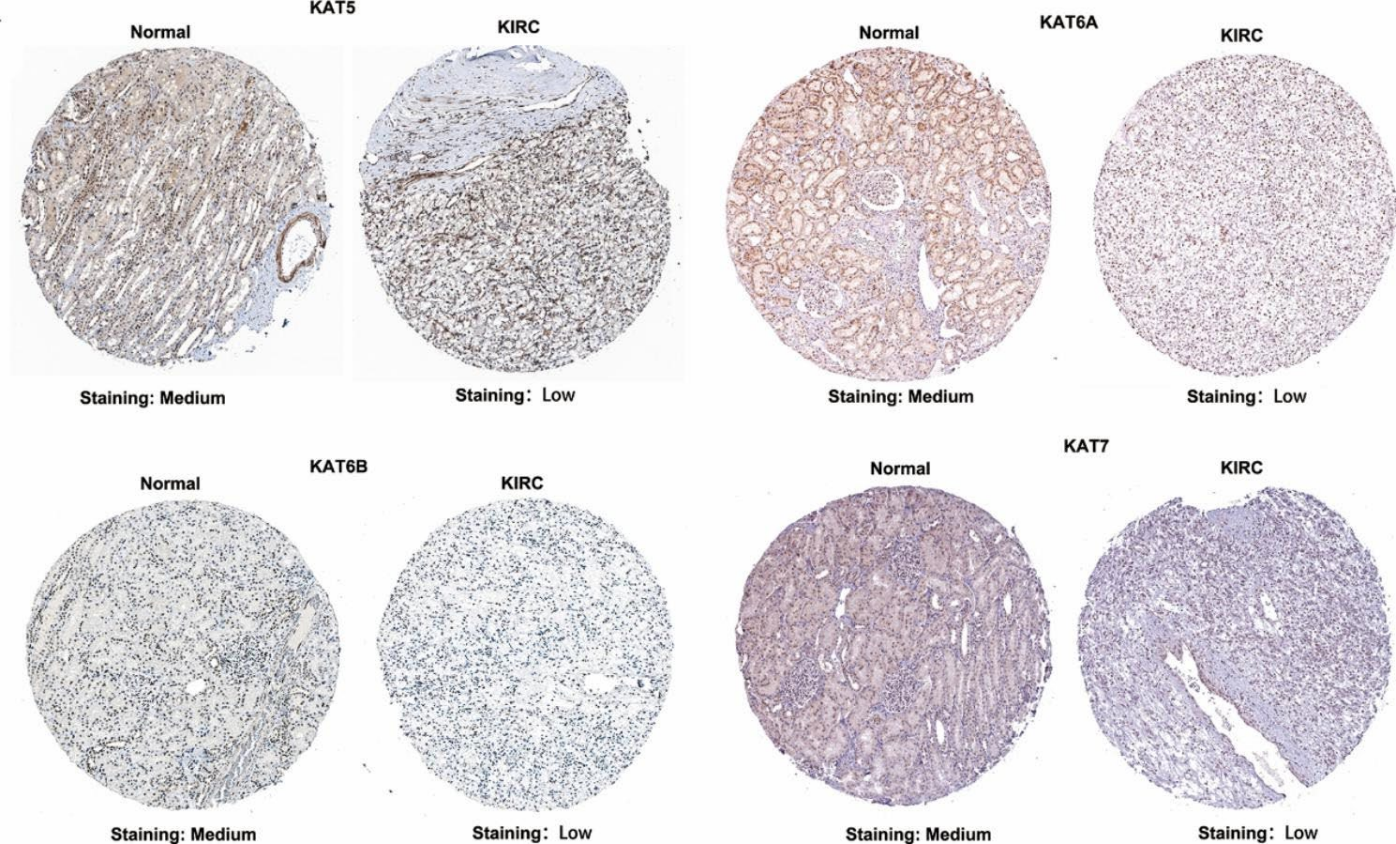
**Immunohistochemical analysis of MYST HATs in KIRC.** The protein levels of MYST HATs in KIRC tissues were compared with those of normal tissues by IHC staining downloaded from Human Protein Atlas

We also performed immunohistochemical analysis on clinical samples from KIRC patients using the Human Protein Atlas database. The immunohistochemical stain results from the database showed that the expression levels of MYST HATs were predominantly in the nucleus (Fig. 4). In KIRC tissues, the expression levels of KAT7 and KAT6B were not detected or at a low level, and the expression levels of KAT5 and KAT6A were at a low or medium level. In contrast, the expression levels of MYST HAT were mainly at a medium level in normal kidney tissue (Fig. 4 and Additional File 3). Our data demonstrated that the mRNA and protein levels of MYST HATs except KAT5 decreased in KIRC.

### The relationship between MYST HATs and their clinical status

**Fig. 5:**
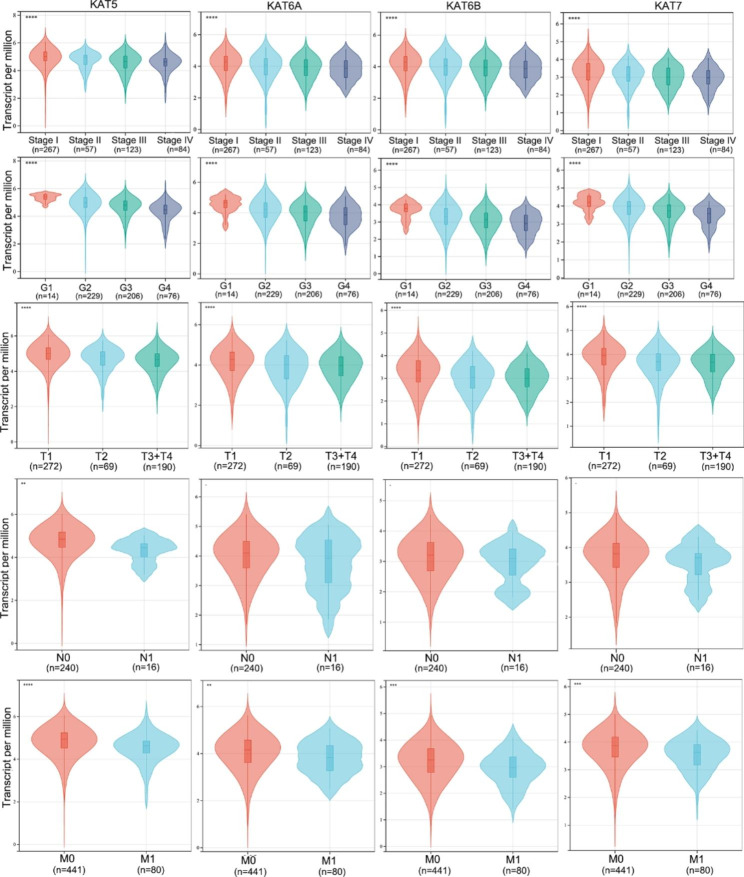
**The relationship between MYST HATs and clinical pathological features.** Expression of MYST HATs in KIRC based on individual tumor stage, tumor grade, T stage, M stage and N stage. **, *P* < 0.01; ***, *P* < 0.001,****, *P* < 0.001. Kruskal-Wallis rank sum test (TCGA database)

**Fig. 6:**
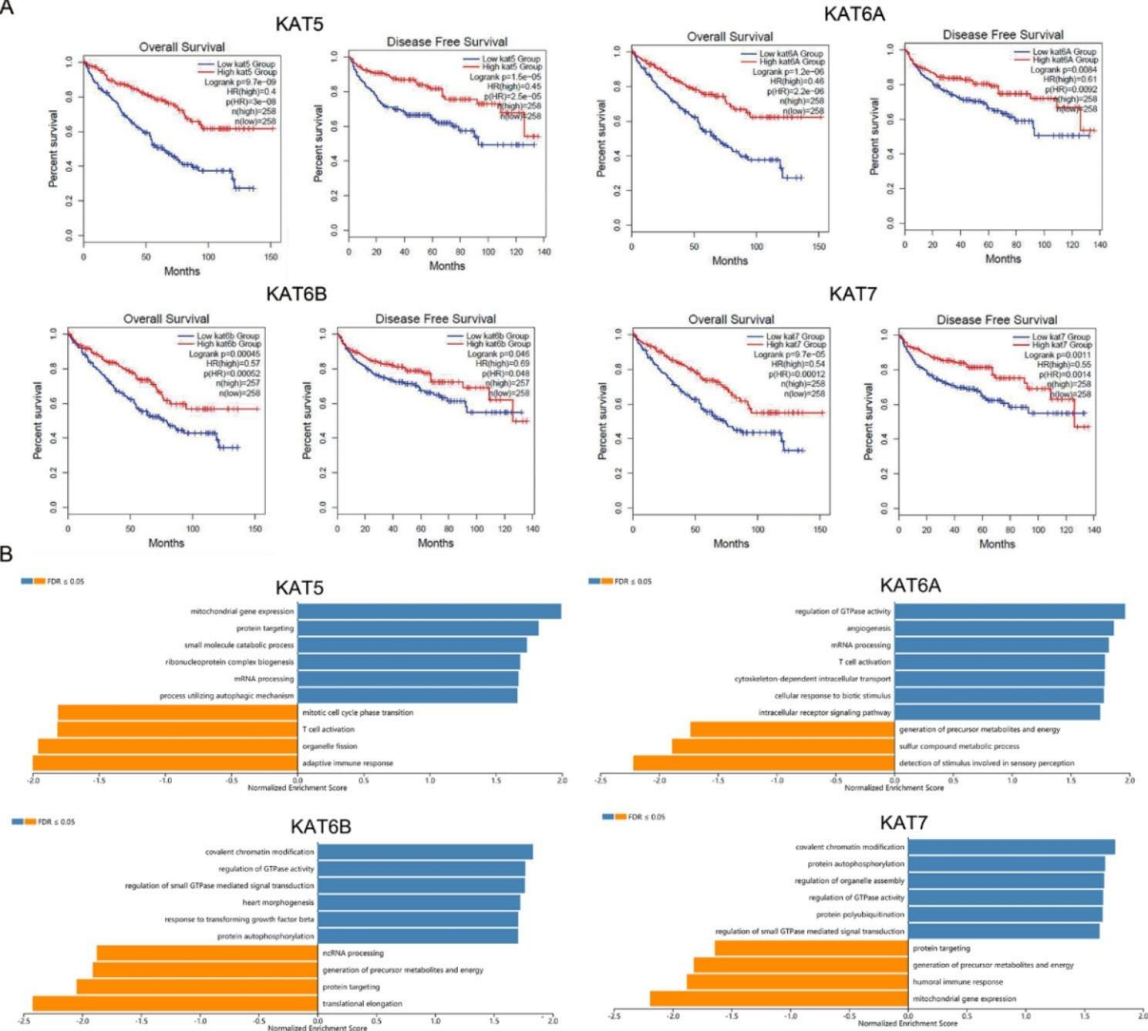
**Kaplan-Meier curve analysis and GSEA analysis based on MYST HAT expression. (A)**Kaplan-Meier curves exhibited the relationships between MYST HAT expression and overall survival or disease-free survival of KIRC patients (GEPIA database). (**B**) GSEA results were performed based on the LinkedOmics database. Pathway alterations in the high expressed group (blue) vs. the low expressed group (orange). FDR < 0.05.

We analyzed the relationship between MYST HATs and clinical pathological features and found that the expression levels of MYST HATs were significantly associated with TNM stage and histological grade (Fig. 5). We also analyzed the relationship between MYST HATs expression and T stage, N stage and M stage. The expression levels of MYST HATs were significantly associated with T stage, and M stage. Only KAT5 were significantly correlated with N stage (Fig. 5). Next, we investigated the prognostic value of MYST HATs in patients with KIRC (Fig. 6A). The Kaplan-Meier curve for overall survival and disease-free survival showed a clear separation between KIRC patients with different expression levels of MYST HATs. Higher mRNA levels of *KAT5*, *KAT6A*, *KAT6B* and *KAT7* were correlated with significantly longer overall survival and disease-free survival (Fig. 5D). All data indicated that MYST HATs could be favorable prognostic indicators for patients with KIRC.

### The predicted functions of MYST HATs in KIRC

GSEA was conducted to explore the potential functions of MYST HATs in KIRC (Fig. 6B). KAT6A, KAT6B and KAT7 were positively correlated with covalent chromatin modification and the regulation of GTPase activity, negatively correlated with the generation of precursor metabolites and energy. KAT6A, KAT6B and KAT7 showed similar functions, but the function of KAT5 was different from that of other MYST HATs. KAT5 was positively correlated with protein targeting and mitochondrial gene expression, and negatively correlated with T cell activation and immune response.

### The expression correlation between MYST HATs and tumor suppressor genes

**Fig. 7:**
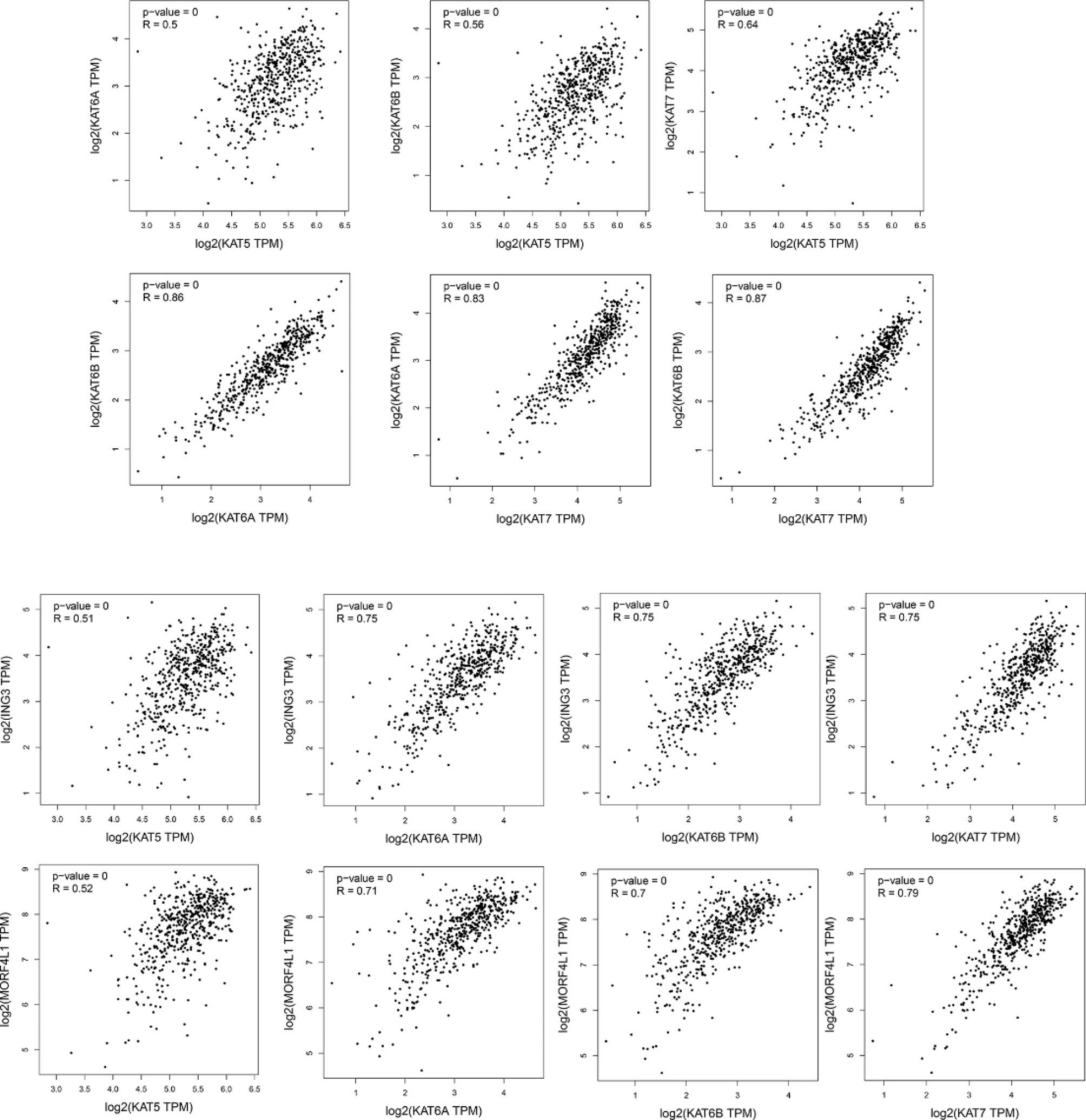
**Expression correlation analysis between MYST HATs and ING3 or MORF4L1.** The analysis was determined using the GEPIA database. TPM (Transcripts Per Million)

We also investigated the expression correlation of MYST HATs with other tumor suppressor proteins in patients with KIRC. MYST HATs function as a protein complex. Additional consideration must be given to the study of the MYST HAT associated protein in the complex. We found that MYST HATs showed a strong expression correlation with each other (Fig. 7). ING3 is a tumor suppressor which is found in multi-subunit complexes formed by all MYST HATs except *KAT8*. All MYST HATs showed a strong positive correlation with ING3 (Fig. 7). MORF4L1 is a member of a gene family related to MORF4 and is involved in cancer cell senescence [15]. MYST HATs showed a strong positive correlation with MORF4L1 (Fig. 7).

### The association between MYST HATs and immune infiltration level

**Fig. 8:**
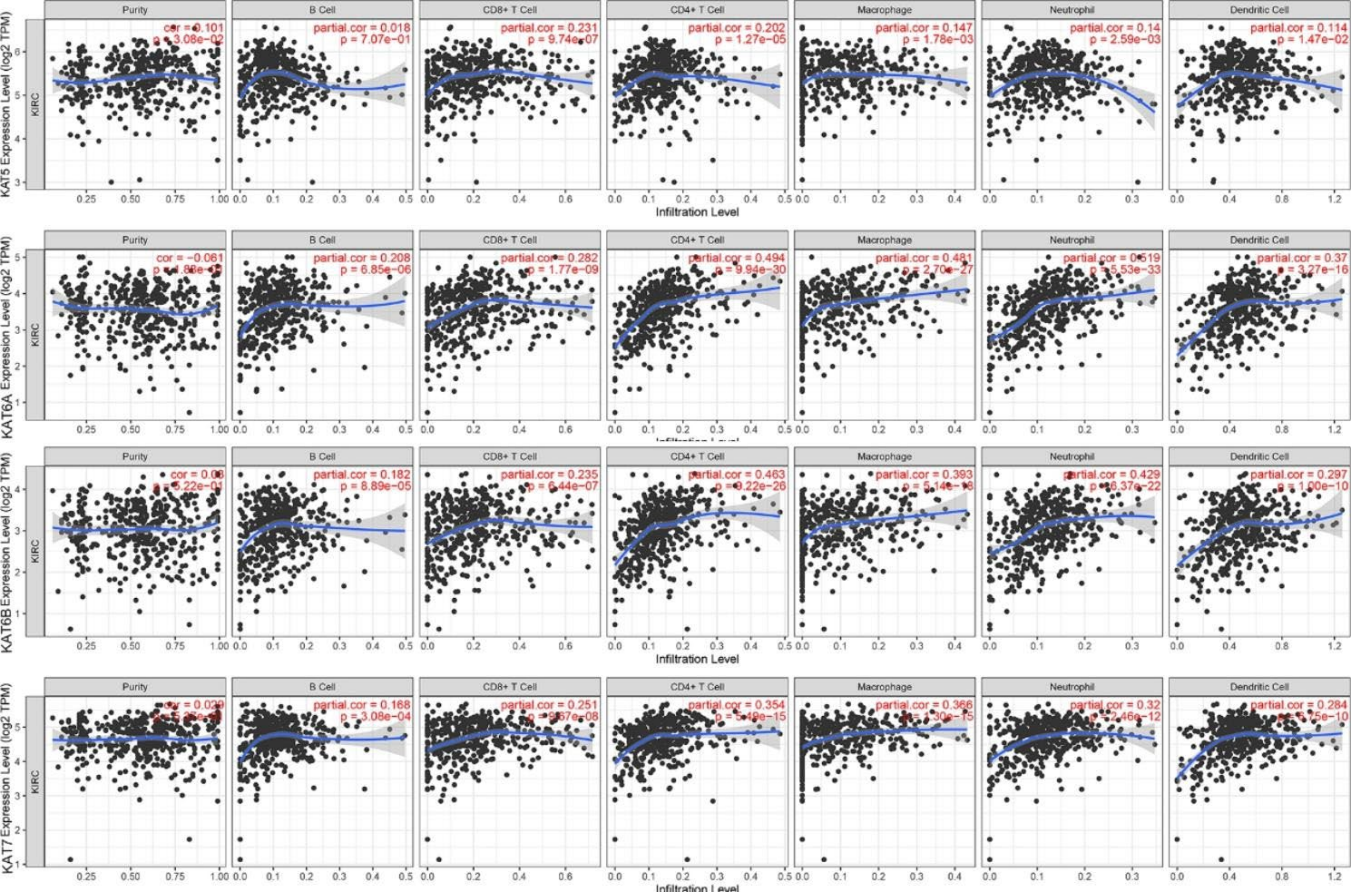
**The correlations between the MYST HAT expression and immune infiltration level in KIRC.** The correlations between MYST HAT and immune infiltrating cells (B cell, CD4^+^ T cells, CD8^+^ T cells, macrophages, neutrophils and DCs) in KIRC were determined using the TIMER database. The levels of gene expression against tumor purity are negative or low, indicating that the purity of gene expression in the tumor is high

Tumor-infiltrating lymphocyteis potentially a new independent predictor of overall survival and sentinel lymph node status in cancer patients. We explored whether the expression of MYST HATs could affect the levels of immune cell infiltration in KIRC. The expression levels of *KAT6A*, *KAT6B*, and *KAT7* showed positive associations with B cells, CD4^+^T cells, CD8^+^ T cells, dendritic cells, macrophages, and neutrophils (Fig. 8), and *KAT6A* exhibited a stronger positive correlation with CD4^+^ T cells, macrophages, and neutrophils than other MYST HATs. The expression of *KAT5* was weakly correlated with tumor infiltrating lymphocytes.

## Discussion

MYST HATs play an important role in transcription regulation, DNA repair, and DNA replication through the acetylation of histone lysine residues [6]. We studied the relationships between MYST HATs and clinical status, and found that MYST HATs except KAT8 are favorable prognostic maker markersfor KIRC patients.

KAT5 plays an important role in genomic instability, gene transcription, and DNA damage repair [16]. Silencing of KAT5 in cells leads to early embryonic lethality [17]. The KAT5 gene is a haplo-insufficient tumor suppressor required for an oncogene-induced DNA damage response [18]. The expression of KAT5 was downregulated in colon, lung, breast, and other cancers [19]. KAT5 and KAT6A were associated with prognosis and tumor mutation burden in KIRC [20]. In our study, low expression of KAT5 predicted advanced TNM stage and was significantly correlated with poor overall survival and disease-free survival in KIRC patients. TCGA data showed that the transcription level of KAT5 in KIRC was higher than that in normal tissues, but the protein level detected by immune histochemistry and western blotting was not significantly different. The mRNA level and the protein level of KAT5 expression exhibited a different trend in KIRC, but the reason was not entirely clear. The gene correlation analysis showed that KAT5 was negatively correlated with T cell activation and the immune response in KIRC. However, TIME database analysis found that KAT5 was weakly correlated with tumor infiltrating lymphocytes. The two types of data explored the relationship between KAT5 and immunity at different levels. Although there were some contradictions, these data indicated that KAT5 could not improve the immune response. In view of some contradictions in the results of KAT5, it is necessary to further study whether KAT5 is a prognostic marker of KIRC.KAT6A and KAT6B have a similar structure with 60% amino acid identity and 66% similarity [21]. Both KAT6A and KAT6B are composed of tandem PHD fingers, a MYST domain, an acid region, and an SM-rich domain [22]. KAT6A and KAT6B play an important role in vertebrate development. The human KAT6A and KAT6B genes mutated recurrently in leukemia, nonhematologic malignancies, and multiple developmental disorders [23]. These two acetyltransferases are considered as good targets for cancer therapy [24]. Inhibition of KAT6A and KAT6B could induce senescence and arrest tumor growth [25]. In our study, the function of KAT6A and KAT6B in KIRC is different from that of other types of tumors. Up-regulation of *KAT6A* and *KAT6B* showed good overall survival benefits in KIRC. We also found that *KAT6A* showed a strong positive correlation with CD4^+^ T cells, macrophages, and neutrophils, which may make it as a favorable marker in KIRC (Fig. 8).

KAT7 complexes are the major acetyltransferase responsible for the histone H4 and H3 acetylation and regulate various cellular functions, such as DNA replication, gene transcription, protein ubiquitination and immune regulation [26]. KAT7 is also involved in cell senescence and is a therapeutic target for ageing [27]. KAT7 is up-regulated in a variety of cancers such as breast, prostate, bladder and gastric cancer [28]. KAT7 is reported to be an anticancer target. Our results are different from previous reports. We found that *KAT7* decreased in KIRC and low expression of *KAT7* was significantly associated with poor overall survival in patients with KIRC. KAT7 also showed positive associations with immune infiltration level.

KAT8 plays an important role in different cell functions, including autophagy, carbon metabolism, gene transcription, DNA damage repair, cell cycle regulation, and early embryonic development [29–32]. KAT8 plays a dual role in the tumor, acting as a suppressor or promoting tumor growth [33–36]. In our study, the high expression of *KAT8* was not related to good overall survival and disease-free survival rate in KIRC (Additional File 4). GESA analysis showed that the function of KAT8 was obviously different from that of other MYST HATs in KIRC.

We also found that the expression of MYST HATs (*KAT5*, *KAT6A*, *KAT6B* and *KAT7*) showed a strong correlation with each other in KIRC. However, the correlation was rare in other cancers. This phenomenon may be related to the special function of MYST HATs in KIRC. MYST HATs function in multiunit protein complexes. We speculated that the antitumor function of MYST HATs may be due to the existence of a tumor suppressor protein in the functional complex. The relationship between MYST HATs and the ING family has been studied extensively. The ING family has been reported as tumor suppressor gene that regulate chromatin function through interaction with histone acetyltransferase or histone deacetylase protein complexes [37]. In our study, ING3 showed a strong correlation with MYST HATs except *KAT8*. It is unclear whether the regulatory effect of *ING3* on MYST HATs can inhibit tumor progression in KIRC. Human MORF4L1(MRG15) is a transcription factor involved in cellular senescence [38]. However, little is known about the role of *MORF4L1* in KIRC. We found that *MORF4L1* showed a strong correlation with MYST HATs except *KAT8*, which implied that MYST HATs may be involved in cellular senescence. More studies were needed to explore the detailed relationship between MYST HATs and *ING3* or *MORF4L1* in KIRC.

Our study has some limitations that need further discussion. First, there is no in-depth study on the relationship between MYST HATs and histone methylation in KIRC. Second, although MYST HATs affect the metastasis and development of KIRC, the specific mechanism has not been explored. Although our analysis is still preliminary and more details need to be improved with experiments, our investigation may help guide a further study of MYST HATs, especially for the role of these genes in immune infiltration.

## Conclusions

We used bioinformatic technology to perform an integrative analysis of MYST HATs and their clinical significance in KIRC based on published TCGA data. Our results indicated that MYST HATs except KAT8 play a beneficial role in KIRC.

## Electronic supplementary material

Below is the link to the electronic supplementary material.

Supplementary Material 1

Supplementary Material 2

Supplementary Material 3

Supplementary Material 4

## Data Availability

The original data of the study are available from the corresponding authors upon reasonable request. The datasets generated and/or analyzed during the current study are available in the GEPIA repository, [http://gepia2.cancer-pku.cn/#index]; in the UCSC Xena repository, [http://xena.ucsc.edu/welcome-to-ucsc-xena/]; in the TIMER repository, [http://timer.comp-genomics.org/]; in the LinkedOmics [http://linkedomics.org/login.php].

## Abbreviations

ACC: Adrenocortical Carcinoma
BLCA: Bladder Urothelial Carcinoma
BRCA: Breast Invasive Carcinoma
CESC: Cervical Squamous Cell Carcinoma and Endocervical Adenocarcinoma
CHOL: Cholangiocarcinoma
COAD: Colon Adenocarcinoma
DLBC: Lymphoid Neoplasm Diffuse Large B-cell Lymphoma
ESCA: Esophageal Carcinoma
HNSC: Head and Neck Squamous Cell Carcinoma
KICH: Kidney Chromophobe
KIRC: Kidney Renal Clear Cell Carcinoma
KIRP: Kidney Renal Papillary Cell Carcinoma
LAML: Acute Myeloid Leukemia
LGG: Brain Lower Grade Glioma
LIHC: Liver Hepatocellular Carcinoma
LUAD: Lung Adenocarcinoma
LUSC: Lung Squamous Cell Carcinoma
MESO: Mesothelioma
OV: Ovarian Serous Cystadenocarcinoma
PAAD: Pancreatic Adenocarcinoma
PCPG: Pheochromocytoma and Paraganglioma
PRAD: Prostate Adenocarcinoma
READ: Rectum Adenocarcinoma
SARC: Sarcoma
SKCM: Skin Cutaneous Melanoma
STAD: Stomach Adenocarcinoma
TGCT: Testicular Germ Cell Tumors
THCA: Thyroid carcinoma
THYM: Thymoma
UCEC: Uterine Corpus Endometrial Carcinoma
UCS: Uterine Carcinosarcoma
UVM: Uveal Melanoma
GSEA: Gene set enrichment analysis
TCGA: The Cancer Genome Atlas.
