# Supplementary material for: Expression profiles and functional prediction of histone acetyltransferases of the MYST family in kidney renal clear cell carcinoma
Source: BMC Cancer. 2023 Jun 26;23:586. doi: 10.1186/s12885-023-11076-x (PMC10291769; doi:10.1186/s12885-023-11076-x)
Supplement: Supplementary file 1 — Supplementary Material 1 [file 12885_2023_11076_MOESM1_ESM.pdf]

**Supplemental Table S1. Patient information**

| Number | Gender | Age | Primary cancer | Pathological classification | T | N | M | Tumor stage [1] |
|---|---|---|---|---|---|---|---|---|
| 1 | female | 44 | Y | KIRC | T2a | N0 | M0 | Stage 2 |
| 2 | female | 72 | Y | KIRC | T2a | N0 | M0 | Stage 2 |
| 3 | female | 66 | Y | KIRC | T2a | N0 | M0 | Stage 2 |
| 4 | female | 57 | Y | KIRC | T2a | N0 | M0 | Stage 2 |
| 5 | female | 73 | Y | KIRC | T2a | N0 | M0 | Stage 2 |
| 6 | man | 62 | Y | KIRC | T2b | N0 | M0 | Stage 2 |
| 7 | man | 71 | Y | KIRC | T2a | N0 | M0 | Stage 2 |
| 8 | female | 57 | Y | KIRC | T2a | N0 | M0 | Stage 2 |
| 9 | female | 66 | Y | KIRC | T2a | N0 | M0 | Stage 2 |
| 10 | man | 57 | Y | KIRC | T2a | N0 | M0 | Stage 2 |
| 11 | man | 59 | Y | KIRC | T2a | N0 | M0 | Stage 2 |
| 12 | man | 56 | Y | KIRC | T2a | N0 | M0 | Stage 2 |
| 13 | man | 53 | Y | KIRC | T2a | N0 | M0 | Stage 2 |
| 14 | man | 57 | Y | KIRC | T3 | N0 | M0 | Stage 3 |
| 15 | female | 48 | Y | KIRC | T3 | N0 | M0 | Stage 3 |
| 16 | female | 66 | Y | KIRC | T3 | N0 | M0 | Stage 3 |
| 17 | man | 65 | Y | KIRC | T3 | N0 | M0 | Stage 3 |
| 18 | man | 61 | Y | KIRC | T3 | N0 | M0 | Stage 3 |
| 19 | female | 67 | Y | KIRC | T1 | N1 | M0 | Stage 3 |
| 20 | man | 49 | Y | KIRC | T2b | N2 | M0 | Stage 4 |

[1] the 7th edition of the AJCC cancer staging manual

KIRC: Kidney Renal Clear Cell Carcinoma
