# Supplementary material for: Expression profiles and functional prediction of histone acetyltransferases of the MYST family in kidney renal clear cell carcinoma
Source: BMC Cancer. 2023 Jun 26;23:586. doi: 10.1186/s12885-023-11076-x (PMC10291769; doi:10.1186/s12885-023-11076-x)
Supplement: Supplementary file 2 — Supplementary Material 2 [file 12885_2023_11076_MOESM2_ESM.pdf]

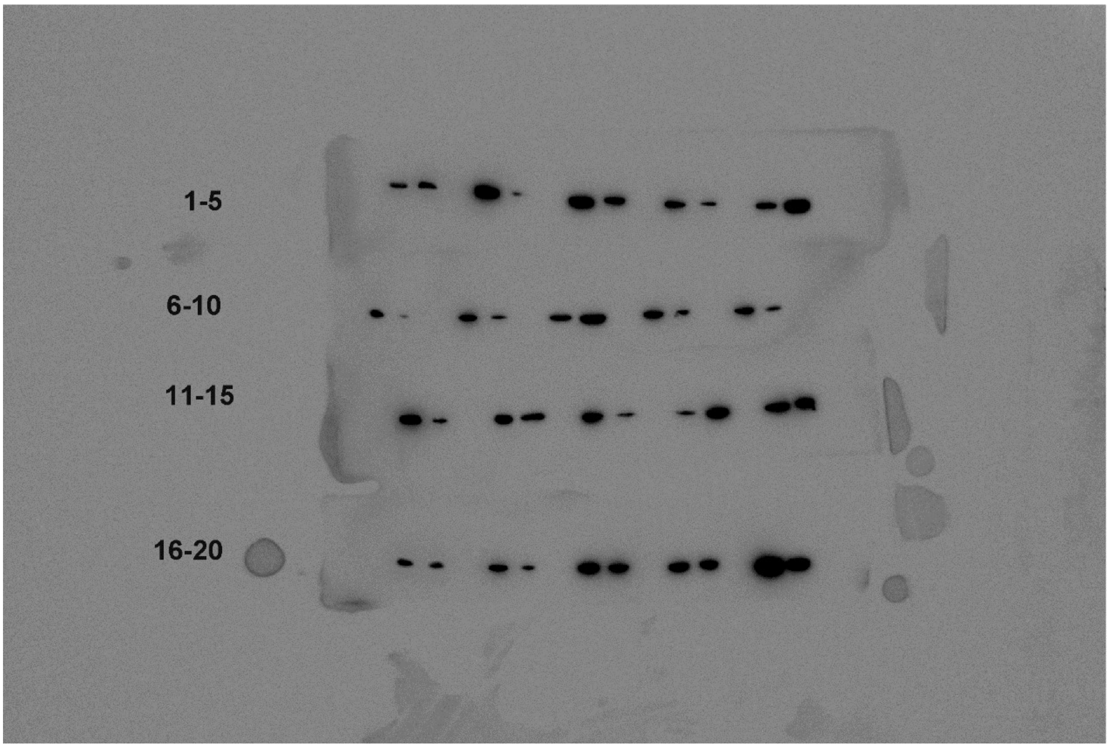

Original western blotting results showing KAT5 protein levels (Figure 3B). After SDS-PAGE, proteins were transferred onto a PVDF membrane. According to the pre-stained protein markers (Thermo Scientific no.26616), the membrane between 40 and 70 kDa region was cut off for immunoblotting with anti-KAT5 antibody (the molecular weight of KAT5 is 58 kDa). By doing so, less anti-KAT5 antibody was used for this experiment. 1-5, 6-10,11-15 and 16-20 samples were on four respective cut PVDF membranes.

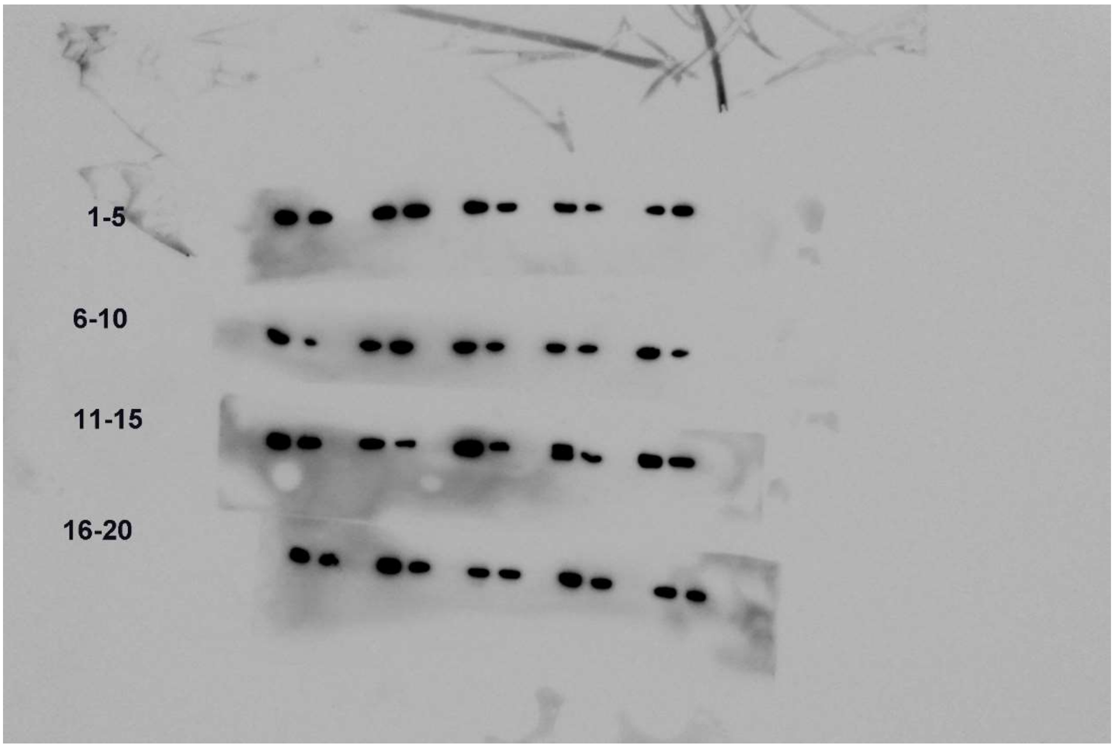

Original western blotting results showing KAT6A protein levels (Figure 3B).After SDS-PAGE, proteins were transferred onto a PVDF membrane. According to the pre-stained protein markers (Thermo Scientific no.26616), >100 kDa region of the membrane was cut off for immunoblotting with anti-KAT6A antibody (the molecular weight of KAT6A is 225 kDa). By doing so, less anti-KAT6A antibody was used for this experiment. 1-5, 6-10,11-15 and 16-20 samples were on four respective cut PVDF membranes.

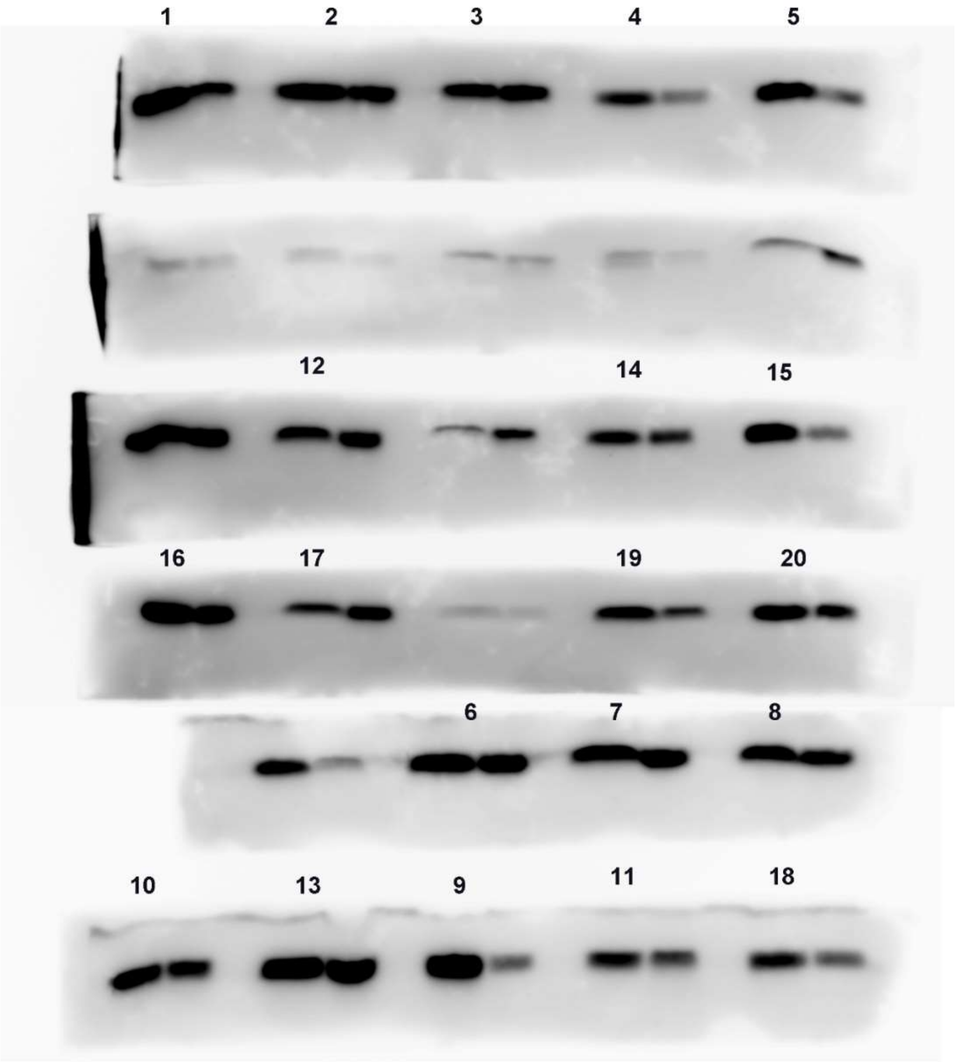

Original western blotting results showing KAT6B protein levels (Figure 3B). After SDS-PAGE, proteins were transferred onto a PVDF membrane. According to the pre-stained protein markers (Thermo Scientific no.26616), >100 kDa region of the membrane was cut off for immunoblotting with anti-KAT6B antibody (the molecular weight of KAT6B is 231 kDa). By doing so, less anti-KAT6BA antibody was used for this experiment.

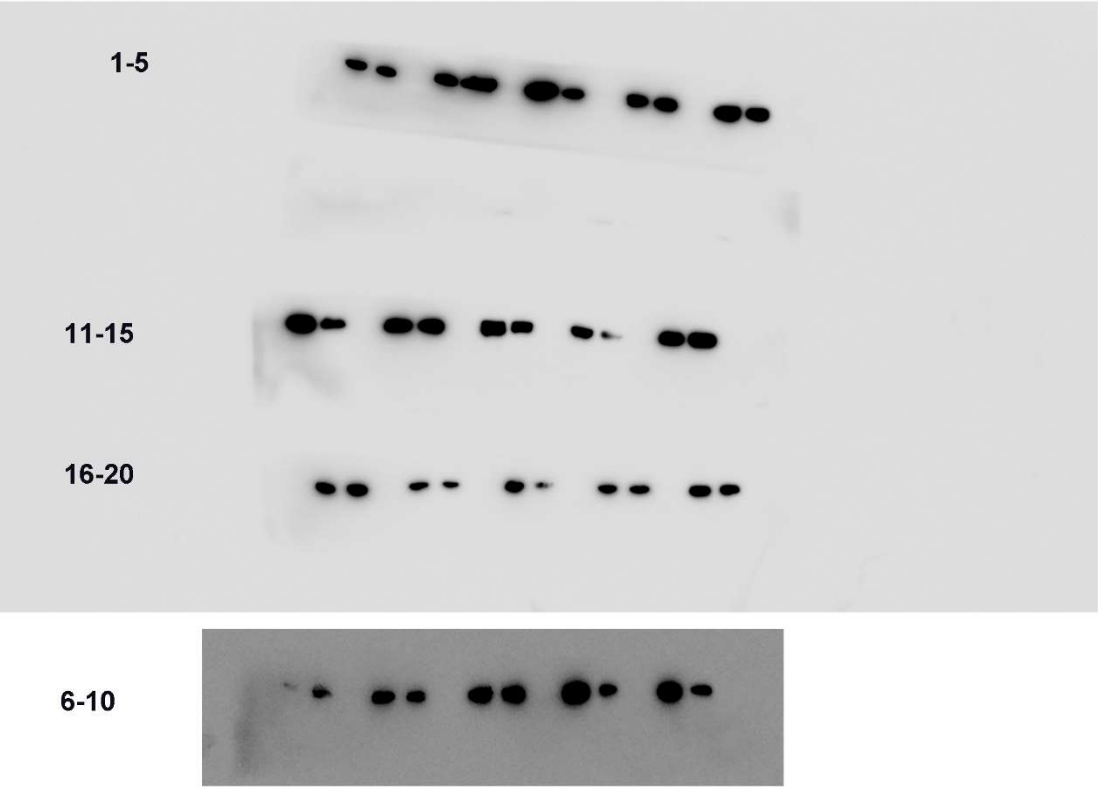

Original western blotting results showing KAT7 protein levels (Figure 3B). After SDS-PAGE, proteins were transferred onto a PVDF membrane. According to the pre-stained protein markers (Thermo Scientific no.26616), the membrane between 55 and 100 kDa region was cut off for immunoblotting with anti-KAT7 antibody (the molecular weight of KAT7 is 71 kDa).

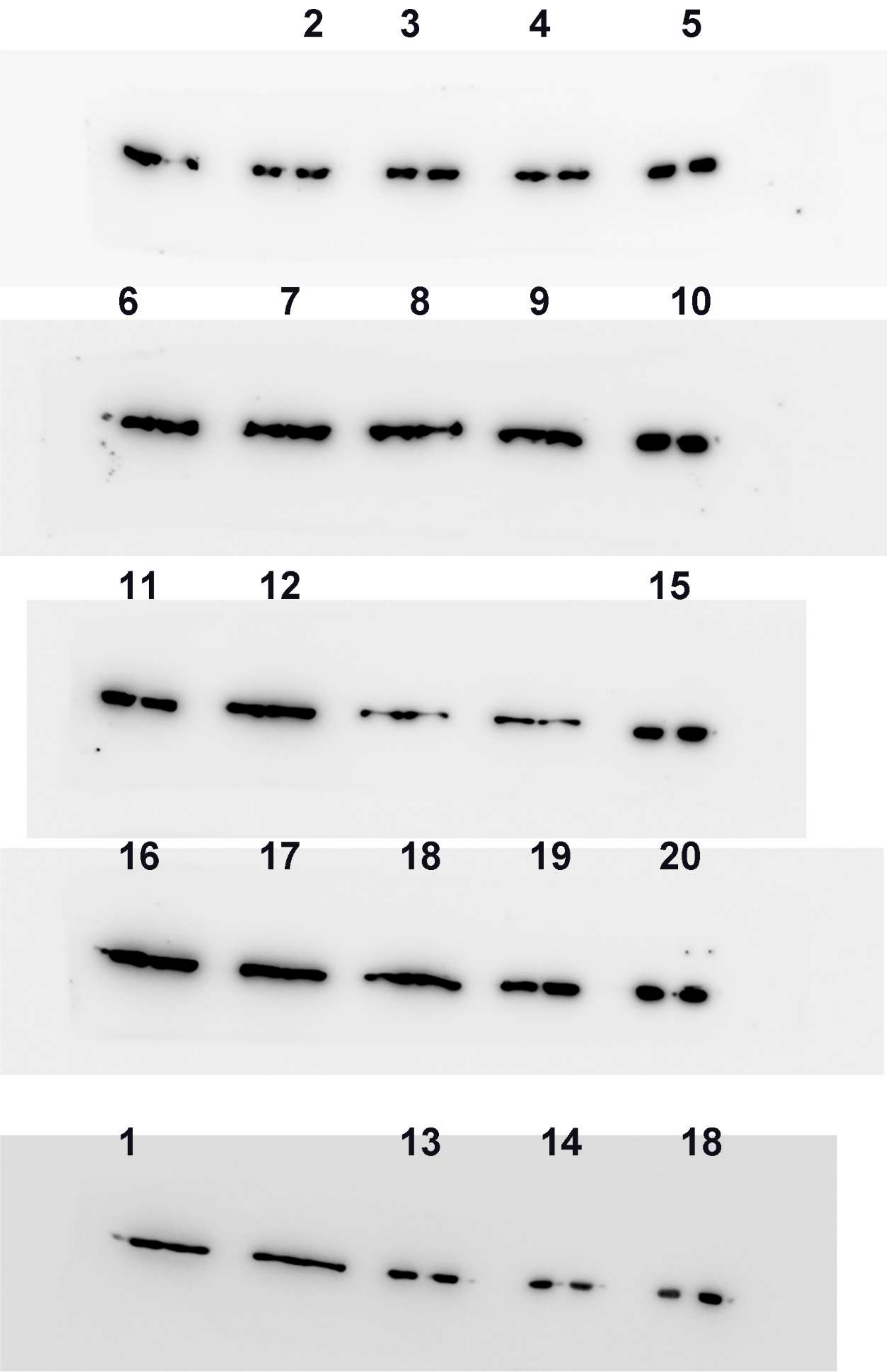

Original western blotting results showing β-actin protein levels (Figure 3B).

After SDS-PAGE, proteins were transferred onto a PVDF membrane.

According to the pre-stained protein markers (Thermo Scientific no.26616), the membrane between 35 and 55 kDa region was cut off for immunoblotting with anti-β-actin antibody (the molecular weight of β-actin is 42 kDa).
