# Supplementary material for: Expression profiles and functional prediction of histone acetyltransferases of the MYST family in kidney renal clear cell carcinoma
Source: BMC Cancer. 2023 Jun 26;23:586. doi: 10.1186/s12885-023-11076-x (PMC10291769; doi:10.1186/s12885-023-11076-x)
Supplement: Supplementary file 3 — Supplementary Material 3 [file 12885_2023_11076_MOESM3_ESM.pdf]

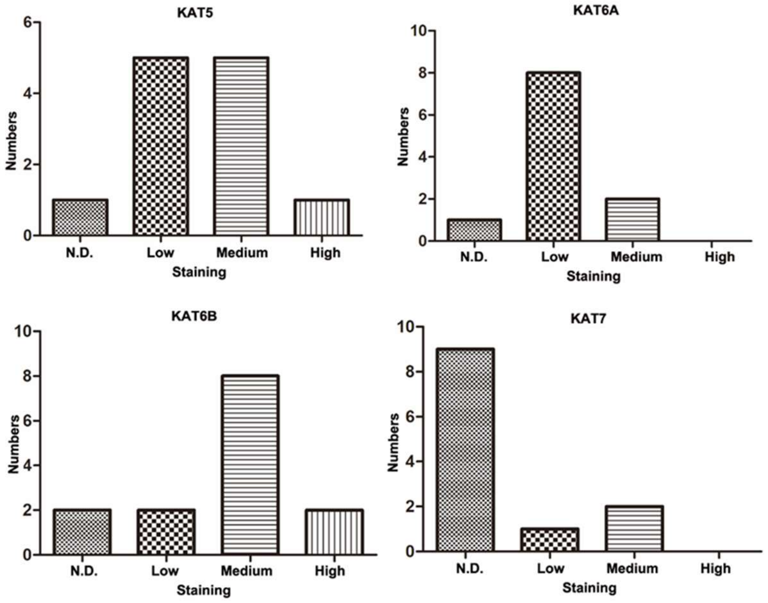

Supplemental The protein expressions of MYST HATs detected by immunohistochemistry The data were downloaded from Human Protein Atlas web. AntibodyHPA063266 for KAT6A; Antibody HPA044470 for KAT7; Antibody HPA006104 for KAT6B; Antibody HPA016953 for KAT5
