# Supplementary material for: Expression profiles and functional prediction of histone acetyltransferases of the MYST family in kidney renal clear cell carcinoma
Source: BMC Cancer. 2023 Jun 26;23:586. doi: 10.1186/s12885-023-11076-x (PMC10291769; doi:10.1186/s12885-023-11076-x)
Supplement: Supplementary file 4 — Supplementary Material 4 [file 12885_2023_11076_MOESM4_ESM.pdf]

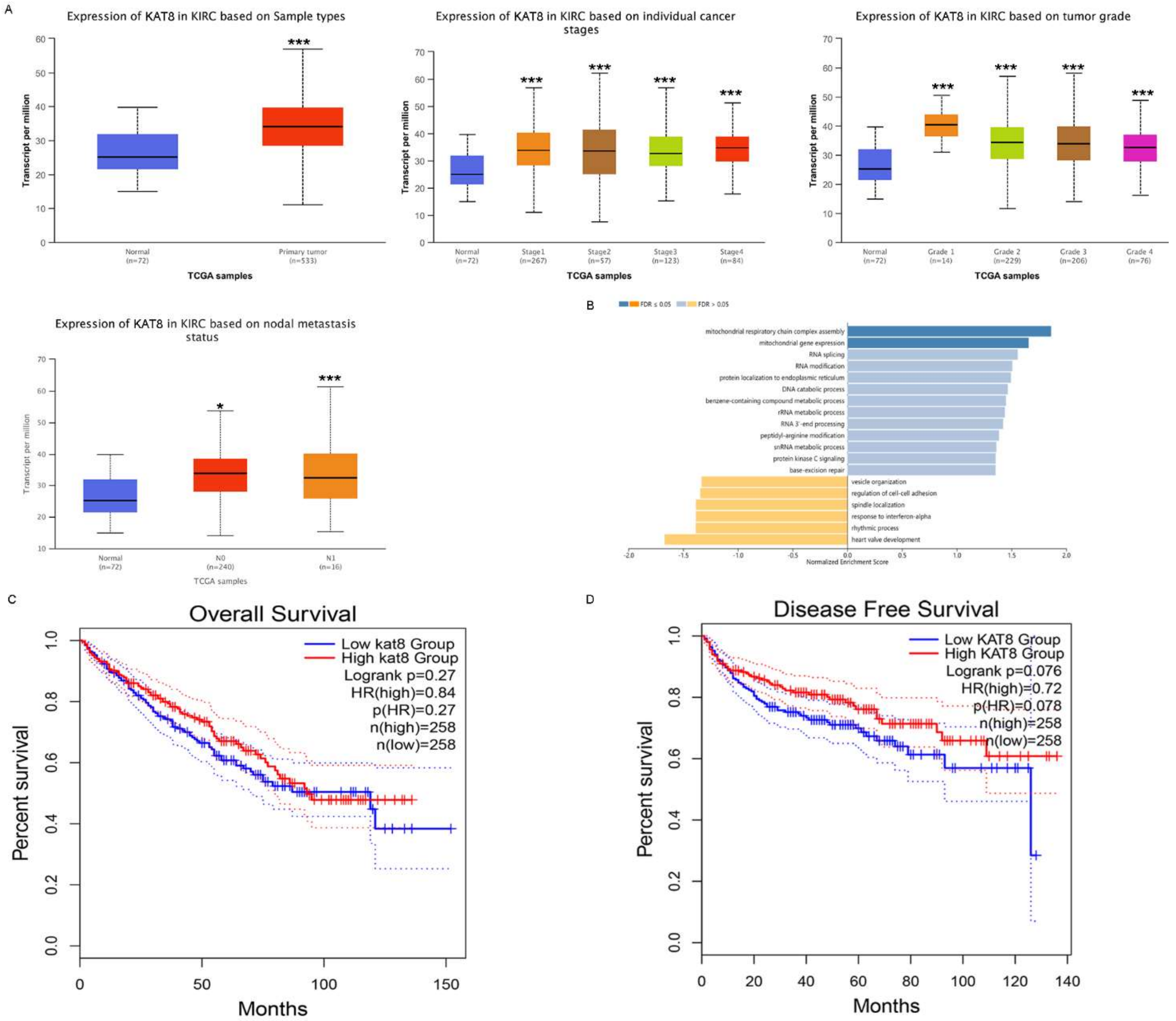

Supplemental Prognostic significance of KAT8 in patients with KIRC (A)Expression of KAT8 in KIRC based on individual cancer stages, metastasis status and tumor grade. *, *P*<0.05; **, *P*<0.01; ***, *P*<0.001. Compared with to normal tissues (UALCAN database). (B) GSEA analysis of KAT8. Kaplan-Meier curves exhibited the relationships between MYST HAT expression and (C) overall survival or (D) disease-free survival of KIRC patients (GEPIA database).
